# Stratification of the risk of bipolar disorder recurrences in pregnancy and postpartum

**DOI:** 10.1192/bjp.2018.92

**Published:** 2018-09

**Authors:** Arianna Di Florio, Katherine Gordon-Smith, Liz Forty, Michael R. Kosorok, Christine Fraser, Amy Perry, Andrew Bethell, Nick Craddock, Lisa Jones, Ian Jones

**Affiliations:** 1 Institute of Psychological Medicine and Clinical Neurosciences, Cardiff University, UK and Department of Psychiatry, University of North Carolina at Chapel Hill, USA; 2 Department of Psychological Medicine, University of Worcester, UK; 3 Institute of Psychological Medicine and Clinical Neurosciences, Cardiff University, UK; 4 Department of Biostatistics, University of North Carolina at Chapel Hill, USA; 5 Institute of Psychological Medicine and Clinical Neurosciences, Cardiff University, UK; 6 Department of Psychological Medicine, University of Worcester, UK; 7 Institute of Psychological Medicine and Clinical Neurosciences, Cardiff University, UK; 8 Department of Psychological Medicine, University of Worcester, UK; 9 Institute of Psychological Medicine and Clinical Neurosciences, Cardiff University, UK and Department of Psychological Medicine, University of Worcester, UK

## Abstract

**Background:**

Pregnancy and childbirth are a period of high risk for women with bipolar disorder and involve difficult decisions particularly about continuing or stopping medications.

**Aims:**

To explore what clinical predictors may help to individualise the risk of perinatal recurrence in women with bipolar disorder.

**Method:**

Information was gathered retrospectively by semi-structured interview, questionnaires and case-note review from 887 women with bipolar disorder who have had children. Clinical predictors were selected using backwards stepwise logistic regression, conditional permutation random forests and reinforcement learning trees.

**Results:**

Previous perinatal history of affective psychosis or depression was the most significant predictor of a perinatal recurrence (odds ratio (OR) = 8.5, 95% CI 5.04–14.82 and OR = 3.6, 95% CI 2.55–5.07 respectively) but even parous women with bipolar disorder without a previous perinatal mood episode were at risk following a subsequent pregnancy, with 7% developing postpartum psychosis.

**Conclusions:**

Previous perinatal history of affective psychosis or depression is the most important predictor of perinatal recurrence in women with bipolar disorder and can be used to individualise risk assessments.

**Declaration of interest:**

None.

Women with bipolar disorder face difficult reproductive decisions. Both clinical and epidemiological studies have found that they have a high risk of recurrence after childbirth.^1^^–^^4^ At the same time, pharmacological treatment may bear teratogenic and possible long-term effects on the offspring and does not guarantee against a recurrence.^4^^,^^5^ Women find it very difficult to get information about the risks involved^6^ and in a survey conducted in the USA, 45% of 70 women with bipolar disorder had been advised by a clinician to avoid pregnancy.^7^ Although the high risk of any postpartum episode for women with bipolar disorder is well established, there is less data to establish the specific risk for individual women. In particular, there is paucity of evidence on the impact of previous perinatal history to guide women with bipolar disorder contemplating further pregnancies.^8^

Our overarching aim was therefore to provide data from which a more individualised assessment of risk can be made to help women and their clinicians. We sought to quantify the risk of a recurrence associated with a second pregnancy and, in particular, the impact of an episode of affective psychosis or non-psychotic depression associated with her first pregnancy.

## Method

**Fig. 1:**
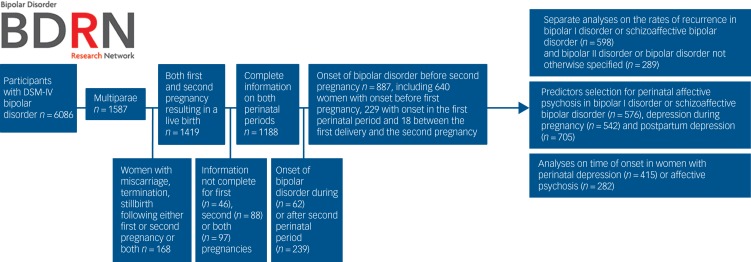
Participant selection and analytic plan.

Figure 1 describes sample selection and the analytic plan.

### Participant recruitment and selection

Women were recruited via systematic (25.7%) and non-systematic (74.3%) methods as part of the Bipolar Disorder Research Network project (www.bdrn.org). Women recruited systematically had been identified through the screening of records of community mental health teams and invited to participate. Non-systematic recruitment included advertisements in general practitioner surgeries and the media, the engagement of patient support organisations and referrals from treating physicians. Women with schizoaffective disorder were significantly more likely than women with other DSM-IV bipolar subtypes^9^ to be systematically recruited (χ^2^ = 21.0063, d.f. = 3, *P* = 0.0001). Apart from diagnosis, no differences emerged in demographic and clinical variables between systematically and not systematically recruited women (Supplementary Table 1 available at https://doi.org/10.1192/bjp.2018.92).

Inclusion criteria for the current study were: (a) a lifetime diagnosis of DSM-IV bipolar disorder (type I, type II or not otherwise specified) or schizoaffective disorder, bipolar type; (b) multiparous women with a first and second pregnancy followed by live births; (c) onset of bipolar disorder before or concomitant with the first delivery. Women who were biologically related to another participant were excluded, in order to include only independent observations. This study received all necessary multiregion and local research ethics approvals by the West Midlands Multi-Centre Research Ethics Committee and participants provided written informed consent.

### Assessment

Women were interviewed using a semi-structured diagnostic interview, the Schedules for Clinical Assessment in Neuropsychiatry.^10^ A life chart was completed and detailed information about family history of psychiatric disorders was collected. Women were also asked to complete a number of questionnaires and permission to review case notes was granted.

Based on the information gathered from the interviews, case-notes reviews and questionnaires, a best-estimate lifetime diagnosis was made according to DSM-IV and key clinical variables were rated. Interrater reliability was formally assessed using a randomly selected sample of 20 participants. The mean kappa statistic was 0.85 for DSM-IV diagnosis and ranged between 0.81 and 0.99 for other categorical variables; mean intraclass correlation coefficients were between 0.91 and 0.97 for continuous variables.

Obstetric history and information on psychiatric episodes occurring in pregnancy and within 1-year postpartum were also obtained from the interview and case notes. Despite the fact that current classification systems limit the postpartum period to 4–6 weeks after delivery, in the current analyses we examined episodes with onset during pregnancy and within 6 months of delivery, to reflect the clinical, everyday definition of the perinatal period.^11^ Affective psychosis was defined as an episode of DSM-IV mania, psychotic depression or mixed episode and depression defined as an episode of DSM-IV non-psychotic depression. We decided not to include perinatal hypomania in the analyses, because of the difficulties in assessing the clinical relevance of hypomanic symptoms in the postpartum period and the validity and reliability of a retrospective account. Indeed, mild postpartum hypomania may be adaptive in some women and in a prospective longitudinal study 11% of 207 women without a clinical history of bipolar disorder reported significant hypomanic symptoms (Highs Scale scores >7) 1-week postpartum.^12^

### Analytic plan

The conditional probability of a psychiatric outcome in the second perinatal period given the outcome of the first perinatal period was calculated using contingency tables. Simultaneous confidence intervals for the conditional probabilities were calculated using the method proposed by Sison & Glaz^13^ and α = 0.95 with the function MultinomialCI in R.^14^ We explored the extent of agreement between perinatal psychiatric outcomes using Cohen's kappa statistics for agreement and its confidence intervals.

As we were interested in personalising the prediction of the psychiatric outcome of the second perinatal period, we also explored the importance of a number of other clinical predictors, beside previous perinatal history. Predictors were chosen from the available data on the basis of the current literature and included: family history of bipolar disorder or perinatal mood disorders, history of childhood sexual abuse and emotional or physical abuse, lifetime comorbidity with panic attack disorder or generalised anxiety disorder, lifetime history of alcohol or substance use disorders, lifetime history of psychosis, number of episodes per year of illness, lifetime history of rapid cycling, prevalent polarity of lifetime mood episodes, DSM-IV diagnosis and whether the onset of bipolar disorder coincided with the first delivery and time interval between pregnancies. Missing data were imputed with the mice package in R. Outcome variables were (a) perinatal affective psychosis in women with bipolar I disorder or schizoaffective disorder, bipolar type and (b) perinatal non-psychotic depression in the whole sample.

We used two different approaches to variable selection: backwards stepwise logistic regression and reinforcement learning trees.^15^ We finally assessed the agreement between times of onset of perinatal episodes separately in (a) women with a history of non-psychotic depression during both the first and second perinatal period and in (b) those with episodes of affective psychosis in relation to both pregnancies. Only women for whom we had complete information on the week of onset of the episodes were included in the analyses. As times of onset were not normally distributed, we used Spearman rank correlations.

The function mosaic in the vcd package was used to create mosaic plots (cran.r-project.org/web/packages/vcd/index.html) and ggplot in ggplot2 (http://docs.ggplot2.org/current/) to plot the variation in the time of onset between a first and second perinatal episode within woman.

## Results

Information on first and second pregnancy resulting in live births was available for 887 multiparous women with bipolar disorder. The demographic and clinical characteristics of the participants are shown in Table 1. Because our recruitment focused on more severe episodes of bipolar disorder, women affected by bipolar I disorder were overrepresented compared with rates of the bipolar subtypes in the general population (estimated lifetime prevalence of bipolar I and II disorder in the general population 0.6 and 0.4%, respectively).^16^

**Table 1:** Sample characteristics

Characteristic		*n*
Age at interview, years: median (minimum, maximum)	48 (19, 85)	
Age at onset, years: median (minimum, maximum)	19 (4, 48)	
Interval between first and second delivery, years: median (minimum, maximum)	3 (0, 16)	
DSM-IV diagnosis, *n* (%)		887
Bipolar I disorder	576 (64.9)	
Bipolar II disorder	262 (29.5)	
Bipolar disorder not otherwise specified	27 (3.0)	
Schizoaffective disorder, bipolar subtype	22 (2.5)	
Lifetime history, yes: *n* (%)		
Psychosis	474 (62.4)	760
Rapid cycling	202 (33.9)	595
Alcohol use disorders	179 (27.4)	653
Cannabis use disorders	53 (7.8)	683
Other substance use disorders	52 (7.6)	683
Panic attack disorder	128 (22.3)	573
Generalised anxiety disorder	385 (65.2)	590
Family clinical history, yes: *n* (%)		
Family history of bipolar disorder in first-degree relatives	403 (48.1)	837
Family history of perinatal mood disorders in first- and second-degree relatives	85 (13.8)	614
History of childhood abuse, *n* (%)		
Only emotional	6 (0.8)	754
Physical and/or emotional	54 (7.2)	
Sexual and/or physical or emotional	123 (16.3)	
First lifetime episode occurring after delivery, yes: *n* (%)	222 (27.9)	
		797

The first lifetime episode of bipolar disorder occurred in relation to childbirth for 28% (222/797) of women. For women with a history of postpartum affective psychosis this proportion was even higher: 57% (108/190) of them did not have any psychiatric history prior to the postpartum episode.

The overall risk of a recurrence of a mood or psychotic episode in relation to a second pregnancy was 55% (288/519; 95% CI 50.2–60.1) for women with any previous perinatal history and 31% (114/368; 95% CI 26.4–35.9%) for those with no mood or psychotic episode in relation to the first pregnancy (χ^2^ = 51.227, d.f. = 1, *P* < 0.0001).

As women with bipolar II disorder and bipolar disorder not otherwise specified have not experienced manic episodes by definition, further results are presented separately from those of women with bipolar I disorder and schizoaffective disorder, bipolar type. Analyses limited to women recruited systematically led to similar results to the analyses conduced on the whole sample.

### Bipolar I disorder and schizoaffective disorder, bipolar type

The rate of any recurrence in relation to second pregnancy was 55% (205/372, 95% CI 50.0–60.4%) for women with an episode in relation to first pregnancy and 34% (76/226, 95% CI 27.4–39.8%) for those with no history of perinatal episodes (depression or affective psychosis: χ^2^ = 25.184, d.f. = 1, *P* < 0.0001).

In the group of women with a history of perinatal affective psychosis 43% (79/185, 95% CI 35.7–50.7) had a recurrence of affective psychosis and a further 9% (17/185, 95% CI 2.2–17.2%) of non-psychotic depression in the second perinatal period.

In the group of women with a history of perinatal non-psychotic depression 50% (93/187, 95% CI 42.8–57.6) had a further episode of perinatal non-psychotic depression and 9% (16/187, 95% CI 1.6–16.5) experienced an episode of affective psychosis in relation to the second pregnancy (Table 2). Interestingly, even though women with no history of perinatal mood episodes were less likely to have an episode in relation to the second pregnancy, 34% still developed a perinatal mood episode in the second perinatal period, which included 10% (22, 95% CI 4.0–16.2%) with affective psychosis and 24% (54, 95% CI 18.1–30.4%) with depression (Table 2).

**Table 2:** Probability of having an episode in the second perinatal period, given the psychiatric outcome of the first pregnancy in women with bipolar I disorder or schizoaffective disorder, bipolar type

First perinatal period	*n*	%	Second perinatal period	*n*	%	95% simultaneous confidence intervals	
Affective psychosis	185	30.9	Affective psychosis	79	42.7	35.7	50.7
			Non-psychotic depression	17	9.2	2.16	17.17
			No occurrence	89	48.1	41.08	56.09
Non-psychotic depression	187	31.3	Affective psychosis	16	8.6	1.60	16.48
			Non-psychotic depression	93	49.7	42.78	57.66
			No occurrence	78	41.7	34.75	49.64
No occurrence	226	37.8	Affective psychosis	22	9.7	4.00	16.22
			Non-psychotic depression	54	23.9	20.27	35.66
			No occurrence	150	66.4	60.62	72.86

It is clear, therefore, from these findings that the clinical presentation (depression or affective psychosis) of the episode in the first perinatal period influenced the psychiatric outcome of the second pregnancy (agreement between episodes Cohen's kappa 0.29, 95% CI 0.23–0.35, *P* < 0.0001).

When the odds of having a recurrence in the second perinatal period were examined, women with an history of perinatal affective psychosis were six times more likely to develop a recurrence of affective psychosis (odds ratio (OR) = 6.0, 95% CI 3.43–10.90) compared with women without any episode in the first perinatal period. An episode of non-psychotic depression in relation to the first pregnancy significantly but less strongly increased the odds of a non-psychotic depressive recurrence (OR = 3.3, 95% CI 2.10–5.23) but these women were not at a significantly higher risk of an affective psychosis (OR = 1.4, 95% CI 0.64–2.97) in the second perinatal period, compared with women without perinatal psychiatric history.

### Bipolar II disorder and bipolar disorder not otherwise specified

The rate of any recurrence in the second perinatal period was 56% (83/147, 95% CI 49.0–65.2%) for women with an episode of depression or affective psychosis in relation to a previous pregnancy and 27% (38/142, 95% CI 19.7–34.0%) for those with no history of perinatal episode (χ^2^ = 24.975, d.f. = 1, *P* = 0.0001, Table 3).

**Table 3:** Probability of having an episode in the second perinatal period, given the psychiatric outcome of the first pregnancy in women with bipolar II disorder and bipolar disorder not otherwise specified

First perinatal period	*n*	%	Second perinatal period	*n*	%	95% simultaneous confidence intervals	
Affective psychosis	10	3.5	Affective psychosis	2	20.0	0.00	49.14
			Non-psychotic depression	1	10.0	0.00	39.14
			No occurrence	7	70.0	0.50	99.00
Non-psychotic depression	137	47.4	Affective psychosis	3	2.2	0.00	11.05
			Non-psychotic depression	77	56.2	48.17	65.07
			No occurrence	57	41.6	33.58	50.47
No occurrence	142	49.1	Affective psychosis	3	2.1	0	9.27
			Non-psychotic depression	35	24.7	17.60	31.80
			No occurrence	104	73.2	66.18	80.40

The odds of a non-psychotic depressive episode in the second perinatal period were significantly higher in women with a history of non-psychotic depression during the first perinatal period than in those without a perinatal history (OR = 4.0, 95% CI 2.32–6.95).

### Multivariate prediction modelling

Although we found a strong association between the psychiatric outcome of the first and second pregnancies, many other clinical variables are likely to be associated with perinatal recurrences. We therefore investigated how important perinatal history was among other possible clinical predictors using random forests. Separate analyses were conducted to examine variables that influence risk of (a) postpartum affective psychosis, (b) non-psychotic depression with onset in pregnancy, and (c) non-psychotic depression with onset within 6 months of childbirth. For postpartum affective psychosis, analysis was limited to women with bipolar I disorder or schizoaffective disorder but for non-psychotic depression the analysis involved the whole sample. The clinical variables evaluated were: family history of bipolar disorder or perinatal mood disorders, history of childhood sexual abuse and emotional or physical abuse, lifetime comorbidity with panic attack disorder or generalised anxiety disorder, lifetime history of alcohol or substance use disorders, lifetime history of psychosis, number of episodes per year of illness, lifetime history of rapid cycling, prevalent polarity of lifetime mood episodes, DSM-IV diagnosis and whether the onset of bipolar disorder coincided with the first delivery and interval between pregnancies. We obtained the same results with different random seeds.

The receiver operator characteristic areas for the logistic regression models were 0.78 for affective perinatal psychosis, 0.80 for depression in pregnancy and 0.71 for postpartum depression (Supplementary Fig. 1).

The psychiatric outcome of the first pregnancy was the most significant variable associated with the psychiatric outcome of the second pregnancy (logistic regression effect size: 5 for postpartum affective psychosis, 4.9 for depression in pregnancy and 6.5 for postpartum depression; all *P*<0.001). Information on other clinical variables only modestly improved our ability to predict the psychiatric outcome of the second pregnancy (Supplementary Fig. 2). Using logistic regression, we also found statistically significant positive associations between postpartum psychosis and number of episodes per year of illness (β = 0.4070; s.e. = 0.141; *z*-value = 2.893; *P* = 0.004; logistic regression effect size = 1.6 compared with 5.0 for history of postpartum psychosis) and postpartum depression and depression as the most prominent polarity of illness (β = 1.130; s.e. = 0.416; *z*-value = 2.717; *P* = 0.006; logistic regression effect size = 2.8 compared with 7.5 for history of postpartum psychosis).

### Timing of onset

A further important clinical issue is whether the timing of the onset in relation to delivery for a previous perinatal episode is predictive of the timing of onset of a further episode. For example, if a woman has had a previous postpartum episode with a specific time of onset, can we use this knowledge to predict the period of highest risk of a perinatal recurrence. Although in clinical practice this may often be assumed, there is no research to our knowledge that has addressed this issue.

As previously observed,^2^ onset of affective psychosis was almost exclusively (90%, 253/282) within the first 6 weeks after childbirth, whereas the onset of non-psychotic depression was more spread out across the perinatal period, with only 59% (247/415) occurring in the first 6 weeks after childbirth. We tested the hypothesis that there was a correlation between times of onset of perinatal episodes separately in (a) women with a history of non-psychotic depression during both their first and second perinatal period, and (b) those with episodes of affective psychosis in relation to both pregnancies. Only women for whom we had complete information on the week of onset of the episodes were included in the analyses.

In women with two episodes of perinatal depression (*n* = 170), we found an association between the timing of onset of the first and the second episode (Spearman correlation 0.57, *P* < 0.001).

There was considerably less variation in the time of onset of episodes of affective psychosis, with 74% (*n* = 54) of women having both psychotic episodes within the first month after childbirth. We therefore did not calculate the Spearman correlation coefficient for affective psychosis.

## Discussion

In this study we wanted to identify if there are easily obtainable clinical variables that can inform the discussion of risk associated with further pregnancies in women with bipolar disorder.

### Predictors of perinatal recurrence

We examined a wide range of factors that clinicians can easily establish during the consultation with women with bipolar disorder who plan a second pregnancy, from family history to course of illness. Of great interest, a history of perinatal affective psychosis or depression was the most robust factor associated with a further perinatal recurrence. None of the other variables we were able to examine significantly improved our ability to predict the psychiatric outcome of the second pregnancy. We found that 55% of women who experienced an episode of affective psychosis or depression in pregnancy or following childbirth had a recurrence in a subsequent perinatal period, consistent with previous estimates obtained in a number of much smaller studies.^8^

Women with a history of postpartum psychosis have an almost one in two risk of a manic/psychotic recurrence after a subsequent delivery. Although it is established that a history of postpartum psychosis is a major risk factor for developing a second psychotic episode in the postpartum,^17^ previous studies have not investigated the influence of a history of perinatal non-psychotic depression, despite its high prevalence in women with bipolar disorder.^2^ We found in fact that, contrary to our expectations, the risk of having any form of perinatal recurrence was slightly higher in women with a history of non-psychotic perinatal depression than in those with a history of postpartum affective psychosis. Our results therefore emphasise the need in women with bipolar disorder to take into account all previous perinatal episodes, including depression and not to focus exclusively on the most severe episodes of illness. Although women with postpartum psychosis are at the highest risk of developing a further severe postpartum episode, women with bipolar disorder with a history of perinatal depression have in fact the highest rates of any form of recurrence.

### Parous women without history of perinatal illness may still develop a perinatal episode in subsequent pregnancies

There is a lack of information on risk in further pregnancies to women with bipolar disorder who had a first child without psychiatric complications. In clinical practice the risk is often treated as for a first pregnancy in a woman with bipolar disorder, even though the risk may be lower as they have had an opportunity to experience a perinatal episode but remained well. Although confirming the suspicion that these women were at lower risk, we found that the absence of mood episodes in or following the first pregnancy does not guarantee that women will not experience a perinatal episode of illness in relation to subsequent pregnancies. About a third of women will experience some form of perinatal episode in a second pregnancy even if they had no episode of illness following a first pregnancy.

Women who remained well through the first perinatal period did appear to be at lower risk of affective psychosis and interestingly the rates of a subsequent episode of perinatal affective psychosis were similar in women with a history of perinatal depression (9%) and in those without any perinatal history (8%). Although this estimate is much lower than the 44% recurrence rate in women with a history of postpartum psychosis after a first delivery, it is still higher than that observed in the literature in women without a psychiatric history^18^ or in those with a history of other mental disorders.^1^

### Likely presentation and timing of a further episode follow the characteristics of the first perinatal episode

It is not known whether there are specific and separate puerperal triggers for postpartum psychosis and postnatal depression. A previous study on 45 women with bipolar disorder and at least two postpartum episodes reported a complete concordance between the polarities of perinatal episodes.^19^ In our study we found that there was a moderate, but not complete, agreement between the clinical presentation of a first and a second perinatal episode (44% for affective psychosis, 48% for depression and 64% for non-occurrence in bipolar I disorder).

Because of the large sample size, we were also able to compare the timing of onset of perinatal depression in women with both a first and a second delivery affected. Again, we found a moderate agreement, with about one in two episodes of depression starting in the same perinatal week as the previous perinatal episode. Our results may therefore not only inform clinicians and patients on the most likely presentation of a bipolar episode in the perinatal period, but also on the likely time of onset.

### Strengths and limitations

In our research we analysed information from a large data collection of women with bipolar disorder (the largest in the literature), with a detailed description of the perinatal episodes and lifetime history. Data were obtained from multiple sources and interrater variability was formally assessed.

Because of the retrospective design of the study, the lifetime research diagnosis allocated to each woman was that appropriate at the time of assessment not at each pregnancy. Moreover, we could not assess the importance of a number of potentially important variables, such as mood stability in pregnancy, complex longitudinal patterns of illness, medications that were prescribed during the perinatal period and the woman's adherence to treatment. Although there is evidence that lithium reduces the risk of relapse in pregnancy and after childbirth,^4^^,^^5^^,^^20^ data for other widely used medications are sparse.^21^^,^^22^ Previous studies found that rates of recurrence in women taking medication in the perinatal period were lower than in those without prophylaxis, but still high, ranging between 20 and 50%.^4^^,^^5^^,^^21^,^1^^,^^22^ It is however, possible that women with a previous episode of postpartum psychosis are more likely to take prophylactic medication in a subsequent pregnancy and following childbirth than those who did not experience a perinatal episode. We had data on medication at the time of delivery for a subsample of 52 women with bipolar disorder recruited during pregnancy and followed up after childbirth. At the time of delivery, the majority of them was taking a mood stabiliser (28, *n* = 54%); over one-third were not taking any medication (*n* = 20, 38%). Because of the complexity of prescription regimes, adherence to treatment and pharmacokinetics changes in the perinatal period, it was not possible to draw conclusions on the effect of medications.

Despite these limitations, our models fit significantly better than the null models (see Supplementary Fig. 1) and represent progress compared with general information on risk given to women with bipolar disorder.

### Implications

In this study, we have examined the rates of perinatal recurrence in women with bipolar disorder. We found a high risk of a recurrence with further pregnancies. Although the rates were significantly higher in women with previous perinatal psychiatric history, women without such episodes were still at risk of developing perinatal illness. The clinical presentation (affective psychosis *v.* non-psychotic depression and time of onset in relation to delivery) of the first perinatal episode predicted the presentation and onset of subsequent perinatal episodes. Although women with postpartum affective psychosis were at the highest risk of developing a further severe postpartum episode, those with a history of perinatal non-psychotic depression had the highest rates of any mood episode recurrence.

**Fig. 2:**
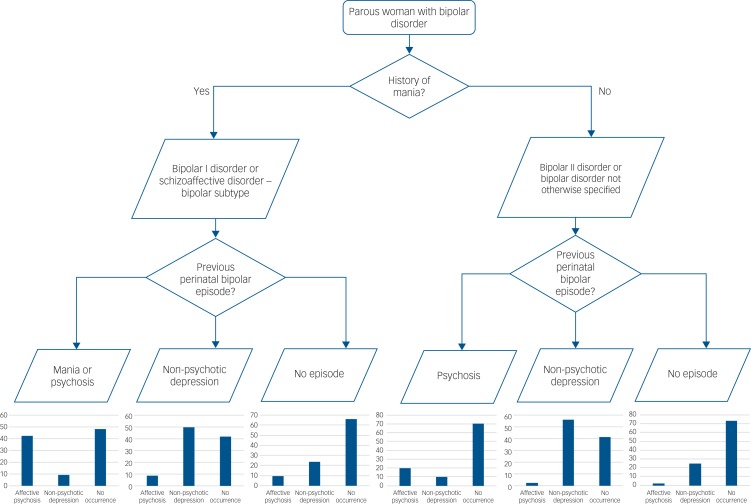
Flow chart for risk assessment of perinatal episodes in women with bipolar disorder who have already had children.

Our study will be of benefit in individualising the risk of perinatal episodes in parous women with bipolar disorder. The risk estimates are summarised in Tables 2 and 3 and a risk assessment flow chart is proposed in Fig. 2 that can be used for preconception and pregnancy counselling. This will help women and their clinicians make the very difficult decisions they face regarding pregnancy.

Hypomania was not included in the analyses, because of the difficulties in assessing the clinical relevance of hypomanic symptoms in the postpartum period and the validity and reliability of a retrospective account.^12^

## Supplementary material

For supplementary material accompanying this paper visit https://doi.org/10.1192/bjp.2018.92. click here to view supplementary material
