# Supplementary material for: Stratification of the risk of bipolar disorder recurrences in pregnancy and postpartum
Source: Br J Psychiatry. 2018 Sep;213(3):542–7. doi: 10.1192/bjp.2018.92 (PMC6429257; doi:10.1192/bjp.2018.92)
Supplement: Supplementary file 1 [file S0007125018000922sup.zip › S0007125018000922sup001.docx]

**Supplemental Figures**

**Supplemental figure 1:** Receiver Operator Characteristic curves of the logistic regression models for A. perinatal affective psychosis, B. depression in pregnancy, C. postpartum depression

A

**
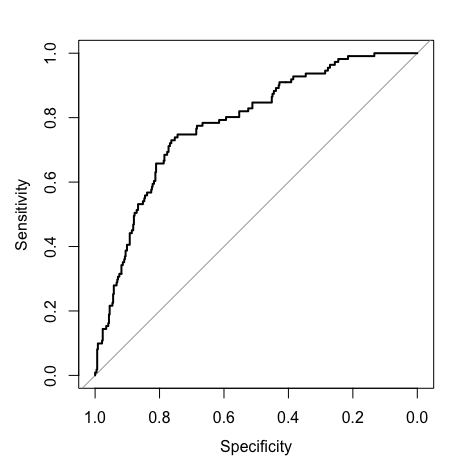
**

**B**

**
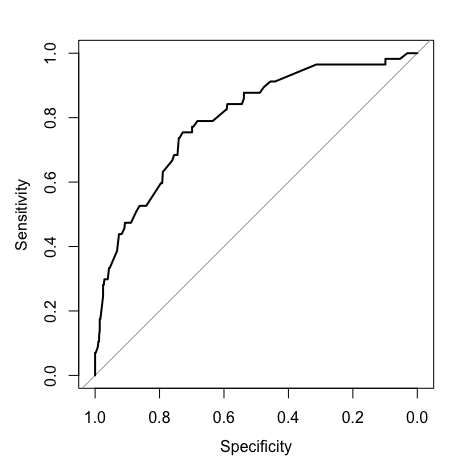
**

**C**

**
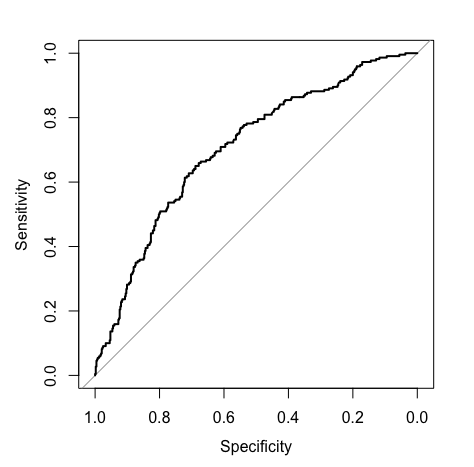
**

**Supplemental figure 2** Variable importance ranked according to the result of classification analyses using reinforced learning trees. Variable importance measure (y-axis) is calculated according to ^1^

Zhu R, Zeng D, Kosorok MR. Reinforcement Learning Trees. *J Am Stat Assoc* 2015; 110: 1770–84.
